# Non-invasive cardiac activation mapping and identification of severity of epicardial substrate in Brugada Syndrome: a case report

**DOI:** 10.3389/fcvm.2024.1304404

**Published:** 2024-01-25

**Authors:** Saverio Iacopino, Paolo Sorrenti, Giuseppe Campagna, Gennaro Fabiano, Emmanuel Fabiano, Jacopo Colella

**Affiliations:** Electrophysiology Unit, Maria Cecilia Hospital GVM Care and Research, Cotignola, Italy

**Keywords:** non-invasive cardiac imaging, Brugada Syndrome, epicardial substrate, case report, Brugada Syndrome ECG type-2 pattern

## Abstract

**Introduction:**

It has recently been shown that electrocardiographic imaging (ECGi) can be employed in individuals undergoing an ajmaline test who have Brugada Syndrome (BrS), to evaluate the extent of substrate-involved arrhythmia in the right ventricular overflow tract (RVOT). For the first time, we stratify the risk of sudden cardiac death (SCD) in BrS during ajmaline testing using the dST-Tiso interval (a robust predictor of the inducibility of ventricular arrhythmias (VAs) in the presence of drug-induced BrS type-1 pattern) in combination with ECGi technology.

**Case presentation:**

We studied a 48-year-old man with BrS ECG type-2 pattern and presence of J-wave without a family history of SCD but with a previous syncope. Transthoracic echocardiography and cardiac magnetic resonance imaging were performed, showing normal results. The ECG was performed to assess the novel ECG marker “dST-Tiso interval.” The 3D epicardial mapping of the RVOT surface was performed with the support of a non-contact cardiac mapping system in sinus rhythm during ajmaline infusion. The examination of the propagation map unveiled the presence of multiple conduction blocks in this pathologic epicardial region, and the conduction blocks were identified within the central part and/or near the boundary separating the normal and slow conduction areas.

**Conclusion:**

The dST-Tiso interval, which lies between the onset and termination of the coved ST-segment elevation and serves as a robust predictor of VA inducibility in cases of drug-induced BrS type-1 pattern, was utilized in conjunction with ECGi technology (employed for the non-invasive confirmation and identification of the pathological substrate area). This combined approach was applied to stratify the risk of SCD in BrS during ajmaline testing, alongside clinical scores.

## 1 Introduction

Brugada Syndrome (BrS) is a hereditary channelopathy linked to an increased risk of developing severe ventricular arrhythmias (VA) and sudden cardiac death (SCD) in individuals who are otherwise healthy (1). Nevertheless, in cases where patients continue to receive recurrent implantable cardioverter-defibrillator (ICD) shocks despite receiving the best available medical therapy, there is an alternative option—radiofrequency transcatheter ablation of the arrhythmogenic substrate. This option has shown promising outcomes (2–4). While there is a general consensus that the pathologic substrate in BrS is primarily located in the right ventricular outflow tract (RVOT) epicardium, the precise origin or pathogenesis of BrS is still a subject of ongoing debate and research. Early theories attributed the propensity for developing VA to abnormal and non-uniform repolarization, leading to concealed phase-two reentry in the epicardium (5); however, more recent evidence suggests that depolarization abnormalities, including slow conduction, conduction blocks, and excitation failure, are thought to have a significant role in arrhythmogenesis in BrS. These abnormalities may result from subtle fibrosis in the RVOT epicardium and abnormalities in gap junctions (6). Sodium channel blockers like Ajmaline, Flecainide, and Procainamide can reveal or exacerbate these alterations, leading to well-documented electrogram (EGM) abnormalities during sinus rhythm. These EGM abnormalities may include low-amplitude epicardial EGMs (less than 1 mV) and/or late potentials (greater than 120 ms) with multiple components (three or more) (3). Today, the identification of complex cardiac arrhythmia sources requires precise substrate mapping, accurate determination of time signal activation, and assessment of propagating wavefronts. Recently, a novel ECG marker known as the “dST-Tiso interval” has been proposed as a predictor of VA inducibility in drug-induced BrS type-I patterns (7). Nonetheless, conventional bipolar signal recording comes with several limitations, and the innovative omnipolar mapping technology (OT) has demonstrated its value as a useful tool in complex mapping (8). OT can be a valuable tool in BrS ablation procedures. It provides immediate assessment and visualization of authentic signal voltage, including its direction and activation speed, with enhanced efficiency and reduced ambiguity compared with the conventional bipolar method. This information can assist in elucidating the disordered signal propagation and wave disruptions in BrS, leading to a clearer distinction between pathological and non-pathological regions when administering sodium channel blockers (9). Recently, it has been demonstrated that electrocardiographic imaging (ECGi) with CardioInsight (non-invasive 3D Mapping System technology, Medtronic Inc.) can be employed in patients with BrS during an ajmaline test to assess the severity of substrate-involved arrhythmia in the RVOT (10). We present a case where, for the first time, a suspected diagnosis of BrS was confirmed by two different non-invasive tests, including the dST-Tiso interval and RVOT activation map analysis using ECGi.

## 2 Case presentation

**Figure 1:**
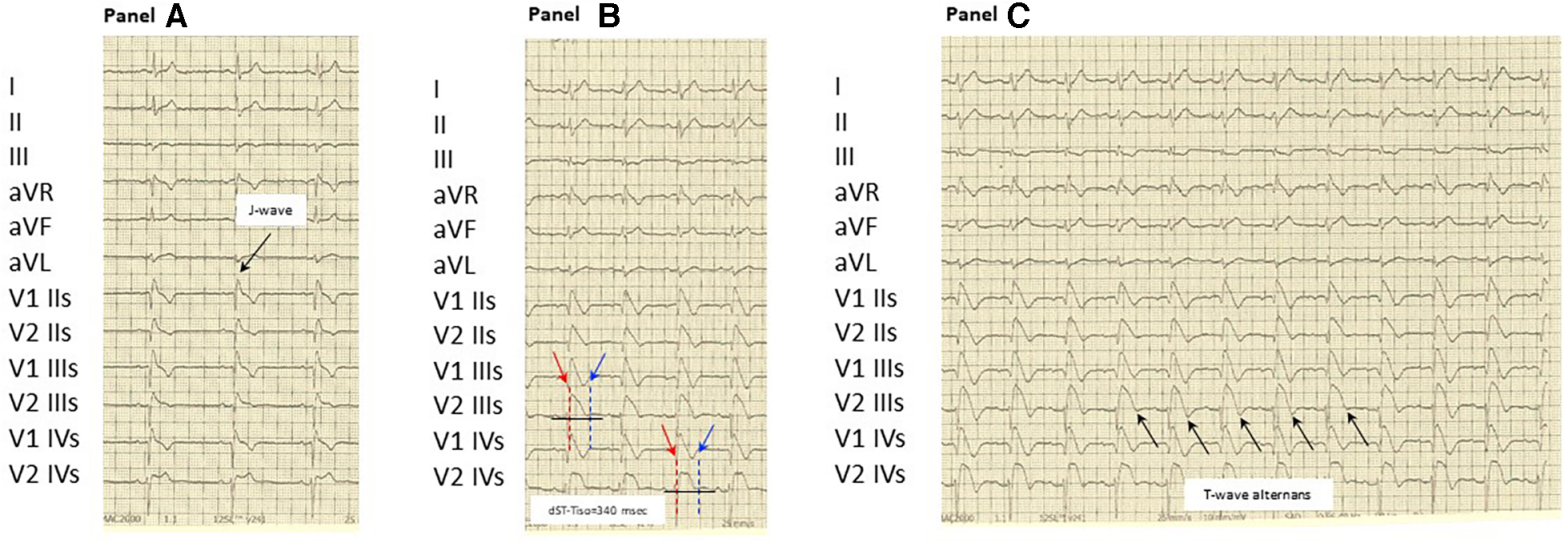
(**Panel A**) At baseline: show the spontaneous type 2 BrS pattern in IV intercostal space (ST-segment elevation of 2 mm in the right precordial leads followed by a convex ST) and presence of J-wave in II intercostal space. (**Panel B**) During ajmaline test: confirming the diagnosis of BrS during ajmaline infusion at 5 min and displaying a dST-Tiso of 340 ms. (**Panel C**) During ajmaline test: showing a T-wave alternans due to alternating activation during ajmaline infusion at 7 min (black arrows). (IIs-IIIs-IVs indicate chest electrodes position in basal condition and during ajmaline infusion: II-III-IV intercostal space). dST-Tiso is obtained measuring the interval between the onset of the coved ST-segment elevation and its termination at the level of the isoelectric line in V2 IIIs and V2 IVs leads.

We studied a 48-year-old man with BrS ECG type-2 pattern and presence of J-wave (Figure 1, Panel A) without a family history of SCD but with a previous syncope. He was admitted to our clinic for risk stratification. In the previous days, during a non-competitive sport medical examination, the ECG occasionally revealed BrS type-2 pattern with J-waves. In the anamnesis, the patient reported repeated episodes of paroxysmal palpitations never documented (but associated with a sense of vertigo) and a syncope. Physical examination and laboratory results were normal. A 24-h Holter monitoring was unremarkable. The 12-lead ECG, with the right precordial leads in the fourth intercostal space, was suggestive of BrS type-2. It showed an ST-segment elevation of 2 mm in the fourth right precordial lead followed by a convex ST. Both transthoracic echocardiography and cardiac magnetic resonance imaging yielded normal results, with no evidence of late gadolinium enhancement in any areas.

## 3 Diagnostic assessment

**Figure 2:**
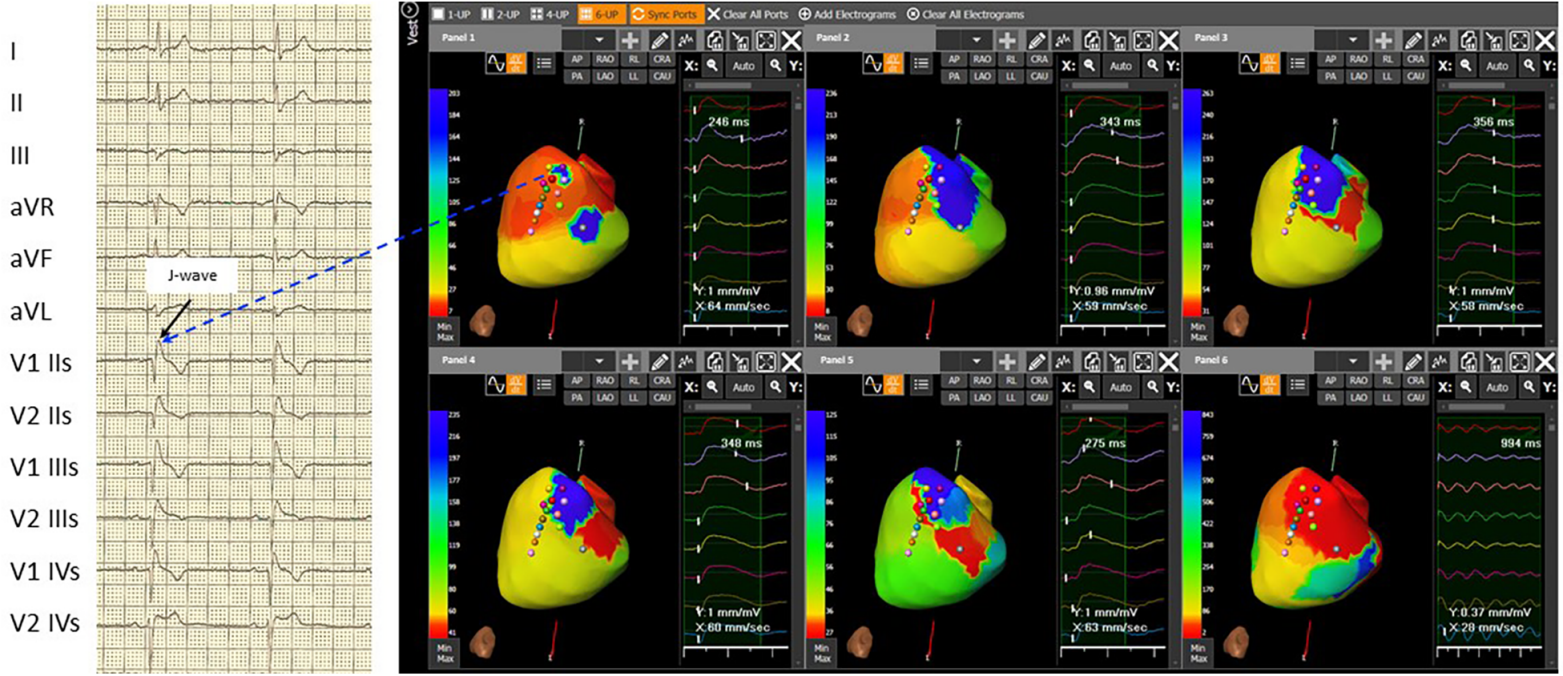
The 3D epicardial RVOT surface was performed with the support of a non-contact cardiac mapping system (CardioInsight) during ajmaline test. In basal conditions, the map shows not late RVOT but only small late areas (related to the presence of J-wave and right delay present on the basal ECG) (**Panel 1**). **Panel 2**, at 5 minutes after the ajmaline infusion, a delay appears on the RVOT in the antero-septal area. **Panel 3**, at 7 minutes after the ajmaline infusion, notice the maximum expression of the Brugada ECG pattern, extension of the delay in the entire anterior area of RVOT, and the appearance of functional block areas. A clear change in color between the later (blue area) and the earliest (red area) is observed in **Panel 4**: at 15 minutes after the ajmaline infusion, there is a reduction of the delayed zone in RVOT and persistence of the functional block area (green line). At 30 minutes after the ajmaline infusion, there is still a reduction of the late area in the RVOT and the disappearance (or clear reduction) of the block areas in **Panel 5**. The activation during induced ventricular fibrillation shown triggering zone (earliest) is depicted in **Panel 6** that coincides with the most late in sinus rhythm in Panel 3. Note: ECG: IIs, second intercostal space; IIIs, third intercostal space; and IVs, fourth intercostal space.

After gaining informed consent, the novel ECG marker denoted “dST-Tiso interval” was assessed and appeared to be 340 ms (Figure 1, Panel B). In our previous study, we demonstrated that the interval between the onset of the coved ST-segment elevation and its return to the isoelectric line in leads V1 and V2 (dST-Tiso interval >300 ms) serves as a robust predictor of VA inducibility when observing the drug-induced BrS type-1 pattern (7). The patient underwent ajmaline test. Also, a T-wave alternans due to alternating activation during ajmaline infusion at 7 min was documented (Figure 1, Panel C). The T-wave morphology in the patient with BrS, exhibiting multiple ajmaline-induced electrogram fluctuations, reveals a notable reliance on the strength of epicardial activation. According to the value of dST-Tiso interval, in order to assess the severity of the substrate, we decided to perform a programmed electrical stimulation (PES) and to study the activation maps using non-invasive ECGi. The 3D epicardial RVOT surface was performed with the support of a non-contact cardiac mapping system (CardioInsight). During the ajmaline test, activation maps were performed in sinus rhythm, under baseline condition (preajmaline infusion), during ajmaline infusion (1 mg/kg in 5 min), and during PES according to the protocols described by Brugada et al. and Priori et al. (11, 12). These protocols consisted of two drive cycles (600 and 400 ms, S1) and three extrastimuli (S2–S4). A minimum coupling interval of 200 ms was established for premature beats in the case of S2 and S3, and it was set to a refractoriness period for S4. During the PES, a VF was induced. At baseline, there were “no areas” exhibiting abnormal EGM readings on the anterior part of the epicardium in the RVOT. However, during ajmaline infusion, a BrS type-1 pattern became evident, and an area of slow activation developed along the anterior wall of the epicardial RVOT. The examination of the propagation map unveiled the presence of several instances of conduction block in this pathological epicardial region, indicating interruptions in electrical conduction. These conduction blockages were observed both in the central part and in close proximity to the border zone between the normal and slow conduction areas (Figure 2, Panels 1–6). Following that, we examined the baseline maps to compare the direction of conduction. It was observed that the line of conduction block that emerged after ajmaline infusion was not present in the baseline maps. It is interesting to observe the comparison between the activation map in the progress of ventricular fibrillation (VF) (Supplementary Video, Panel A) compared with the activation map at 7 min from the ajmaline infusion (Supplementary Video, Panel B). By observation, the 7-min propagation in sinus rhythm confirms the block areas and coincides with an “aborted” reentry mechanism (i.e., with block in entry and exit). In the case of the triggering of VF with three extrastimuli from RVOT, the antegrade activation front finds the same block area followed by a late activation with a reentry mechanism that leads to the VF triggering*.* Ultimately, a genetic test was required to identify the genetic alteration consistent with BrS, and subsequently, the patient successfully underwent transvenous single-chamber defibrillator implantation (SC-ICD). At the 6-month follow-up, the patient was asymptomatic, and infrequent ventricular extrasystole and non-sustained ventricular tachycardia (maximum three beats) were recorded.

## 4 Discussion

In this case report, we stratified the risk of SCD in a patient exhibiting a BrS ECG type-2 pattern. We employed a combination of two non-invasive diagnostic exams—the dST-Tiso interval and ECGi technology—to unveil the presence of abnormal substrate. The outcomes were subsequently validated through invasive exams, including PES and induction of VF. In a brief historical overview, traditional substrate maps based on bipolar signals have been primarily influenced by the direction of a wavefront toward the mapping dipole. These bipolar maps do not supply the essential information needed for a comprehensive characterization of the ablation target. Recently, Porta-Sanchez et al. (13) conducted an evaluation using voltage maps obtained through an equally spaced electrode array and omnipolar mapping (OT) electrogram (EGMs) in the endocardium of 10 pigs. The results indicated that OT EGMs are more effective in distinguishing between infarcted and non-infarcted areas compared with traditional bipolar EGMs. Furthermore, achieving accurate refinement of the pathological borders in the RVOT can be important. Failure to ablate pathological areas could potentially lead to arrhythmic recurrences during the follow-up period (14). The voltage maps obtained with an evenly spaced electrode array and omnipolar EGM technology, used to characterize the epicardial substrate in BrS, better delineate areas of delayed conduction that give rise to wavefront fragmentation and lines of blocks. Utilizing an intracardiac non-contact mapping array and isochronal mapping during a ventricular S1–S2 stimulation protocol, Lambiase et al. discovered a notable conduction delay in the RVOT among individuals with BrS, a phenomenon not observed in healthy controls (14). Furthermore, these areas with delayed conduction exhibited wavefront fragmentation and lines of blockage, ultimately leading to polymorphic VT degenerating into VF in 5 out of 18 BrS patients (14). This arrhythmogenic behavior has also been recently documented by Haïssaguerre et al. (6). In this report, localized conduction blockages occur at multiple sites following a single premature stimulus or during sodium channel-blocker infusion. These blockages could potentially be responsible for the initiation of VF. Moreover, during ajmaline infusion we observed a large spectrum of T-wave morphologies (including monophasic and biphasic patterns) as described by Haissaguerre et al. (15). The morphologies and alternans of T-waves can be associated with abnormal tissue activation. More recently, ECGi has proven to be effective in elucidating the mechanisms underlying a wide range of cardiac arrhythmias, and it is essential to follow an optimal workflow to achieve the best clinical outcomes. In the ECGi procedure, it is crucial to manually verify the signals recorded by the system and included in the calculations to prevent errors associated with automatic processing. Signals of good quality with high amplitude yield maps of the highest accuracy, as demonstrated in our case. In addition, BrS presents as spontaneous variations in electrocardiographic markers, suggesting dynamic changes in the electrical substrate. The use of ECGi methodology in patients with unconfirmed BrS can be highly valuable in the risk assessment process. This is due to its non-invasive nature, making it painless, and its ability to provide additional information about the cardiac substrate and its severity. This remains true even in cases involving evaluations for ablation. When the effectiveness of ECGi methodology is demonstrated by rigorous studies, it may be possible to replace PES study and the induction of VF with the assessment of the ECGi substrate. This could streamline the management of asymptomatic patients with suspected Brugada Syndrome on ECG. Risk stratification for SCD in BrS remains a subject of ongoing debate. As a result, clinical predictors are an attractive and practical solution, especially when incorporated into risk assessment scores.

Sieira et al. (16) have recently introduced a dependable risk score model aimed at predicting the occurrence of SCD in this patient population. Likewise, the Shanghai score has been validated for SCD risk stratification. However, its predictive accuracy has been primarily demonstrated in patients who have not experienced a previous episode of VF (17). In the current guidelines (18) and in clinical practice, there is a lack of consensus on the most appropriate clinical management for patients who exhibit BrS induced by ajmaline. In summary of the data from this case report and supporting studies, the dST-Tiso interval (a robust predictor of VA inducibility in drug-induced BrS type-1 pattern) in combination with ECGi data from the CardioInsight System technology (used to confirm and identify the pathological substrate area non-invasively) could be employed for risk stratification of SCD in BrS during ajmaline testing, alongside clinical scores. Importantly, it is evident that this is a single case review, and the results should not be generalized without further research. Moreover, it is vital to follow the current guidelines (1) to diagnose the entirety of BrS and the subsequent steps, and additional studies with larger cohorts of patients need to be considered to confirm these findings. In particular, randomized studies are needed to assess the value of ECGi to improve the pathway of patients with BrS and to indicate the use of cardiac implantable electronic devices (CIEDs) or provide clear guidance on substrate ablation or, simply, a follow-up strategy.

## 5 Conclusions

This case described the use of two different non-invasive examinations to assess SCD risk. In this case review, both tests revealed an abnormal substrate; however, further large studies may assess the predictive value of a single test vs. the value of combined tests.

## Data Availability

The raw data supporting the conclusions of this article will be made available by the authors, without undue reservation.

## Supplementary material

Supplementary Video Comparison between the map of activation in progress of ventricular fibrillation (VF) (VIDEO, Panel A) compared to the map of activation at 7 minutes from the administration of Ajmaline (VIDEO, Panel B). Note how the 7-minute propagation in sinus rhythm confirms the block areas and coincides with an "aborted" reentry mechanism, that is, with block in entry and block in exit. In the case of the triggering of VF with three extra-stimuli from RVOT; the antegrade activation front finds the same block area followed by a late activation with reentry mechanism that gives rise to the triggering of VF.
